# A live imaging‐friendly slice culture method using collagen membranes

**DOI:** 10.1002/npr2.12128

**Published:** 2020-08-05

**Authors:** Ari Ogaki, Tasuku Araki, Masaya Ishikawa, Yuji Ikegaya, Ryuta Koyama

**Affiliations:** ^1^ Laboratory of Chemical Pharmacology Graduate School of Pharmaceutical Sciences The University of Tokyo Tokyo Japan; ^2^ Isehara Research Laboratory Technology and Development Division Kanto Chemical Co., Inc Suzukawa, Isehara, Kanagawa Japan; ^3^ Center for Information and Neural Networks Suita, Osaka Japan

**Keywords:** hippocampus, imaging, microglia, slice culture, time‐lapse imaging

## Abstract

**Aim:**

Organotypic brain slice culture preserves the geographical position of neurons and neuronal circuits. The slice cultures also maintain both non‐neuronal cell types and the surrounding extracellular matrix. The interface method has been widely used for slice cultures, in which brain slices are placed on semiporous polytetrafluoroethylene (PTFE) membranes. However, a low optical transparency of PTFE membrane makes it difficult to perform live imaging of deep regions of slice cultures using an inverted microscope. To overcome the issue, we evaluated the suitability of using collagen membranes for slice cultures, especially focusing on live imaging of the cellular dynamics of green fluorescent protein (GFP)‐expressing microglia.

**Methods:**

Entorhinohippocampal slices were cultured on either collagen or PTFE membranes. The influence of membrane type on the ability to observe deep regions of slice cultures was examined by live imaging using an inverted microscope.

**Results:**

Collagen membranes were thinner and had better optical transparency compared with PTFE membranes. There were no differences in cell viability, density of neurons or microglia. The densify of visible short branches of microglia in live imaging was higher in collagen membranes than PTFE membranes.

**Conclusion:**

Collagen membranes are suitable for live imaging of cellular dynamics in slice cultures using an inverted microscope.

## Abstract 2

Live imaging of organotypic slice cultures has been a useful method to study cell dynamics. One remaining issue with live imaging of slice cultures is the low transparency of commonly used polytetrafluoroethylene membranes. Here we report that slice culture membranes made of collagen can solve the issue of low transparency, facilitating live imaging of small cellular structures such as microglial processes.

## Graphical abstract

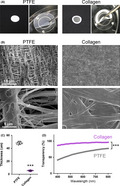

## 1 INTRODUCTION

**FIGURE 1:**
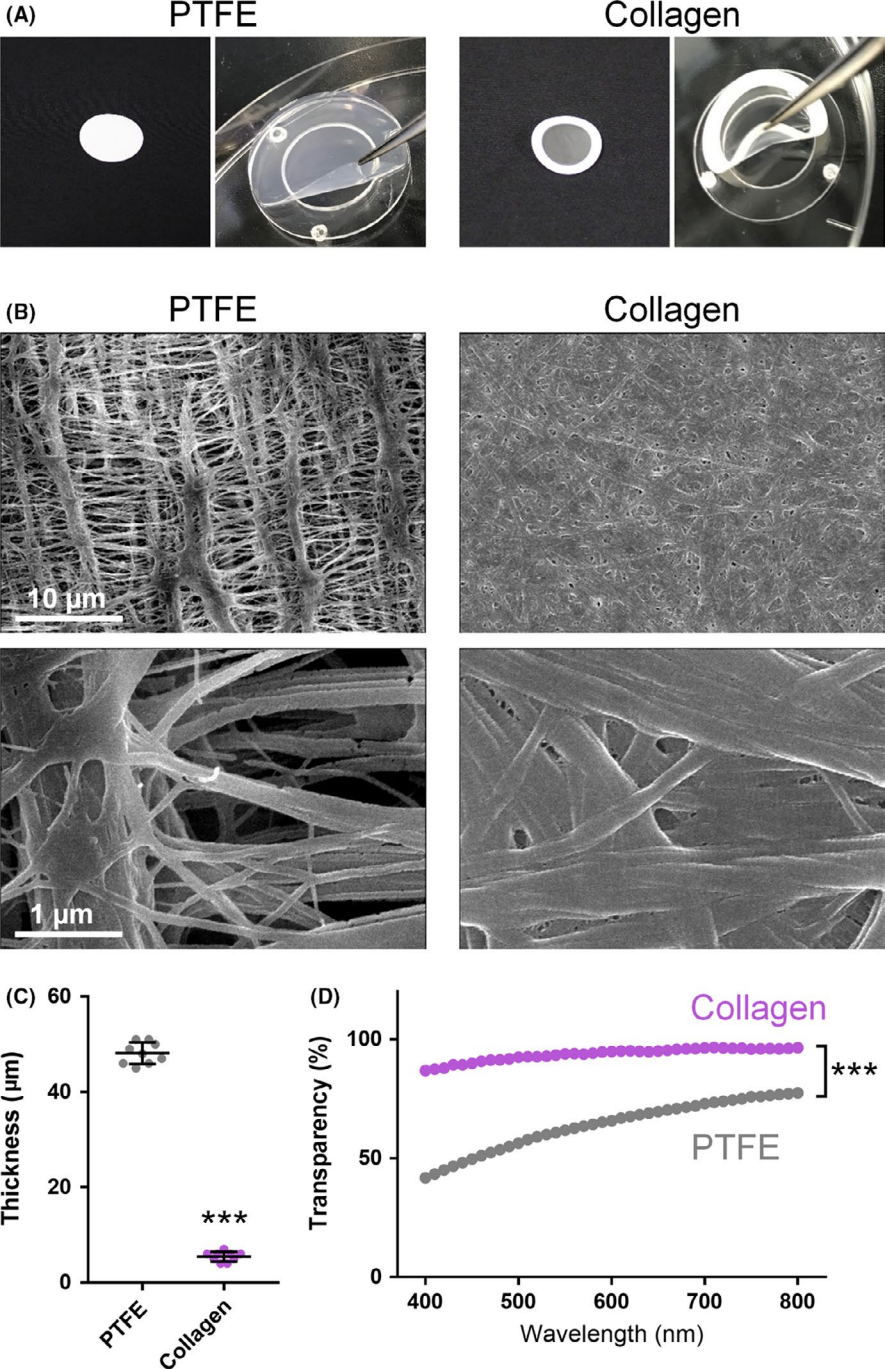
A, Representative images of PTFE and collagen membranes. Membranes were placed in donut plates for slice cultures. B, Representative images of PTFE and collagen membranes using a scanning electron microscopy at 3000× (upper) and 30 000× (lower) magnification. C, Thickness of PTFE and collagen membranes. *** *P* < .001 vs PTFE membranes; Student's *t* test, n = 9 membranes. Data represent mean ± SD. D, The transmittance of PTFE and collagen membranes from 400 to 800 nm. *** *P* < .001 vs PTFE membranes; two‐way repeated‐measures ANOVA followed by Tukey's test, n = 9 membranes. Data represent mean ± SD

Organotypic brain slice cultures are useful for studying interactions among brain cell types, including neurons and glial cells, and for examining the structure and function of neural circuits, which are preserved similar to in vivo when this method is used. Three main methods are used for organotypic slice cultures: (a) roller‐tube cultures, (b) interface cultures, and (c) collagen gel cultures.^1^ The most common brain slice culture method is interface culture in which brain slices are placed on semiporous polytetrafluoroethylene (PTFE) membrane inserts that are resting in medium‐containing tissue culture wells. Our laboratory uses PTFE membranes placed on O‐shaped donut plate inserts for interface brain slice cultures (Figure 1A).^2^

Live imaging of slice cultures enables monitoring of cellular dynamics such as migration, growth of cellular processes, and cell–cell interactions. Such live imaging has classically been performed by either observing cultured slices from above (upright microscope)^3^, ^4^, ^5^, ^6^ or from below (inverted microscope).^7^

Live imaging of slice cultures has been widely conducted using upright microscopes equipped with the water immersion lenses^3^ or the long distance dry objective lenses.^4^, ^5^, ^6^ Meanwhile, when inverted microscopes are used for live imaging, the observation of deep regions of slice cultures is difficult because the distance between the objective lens and the slice usually surpasses the range of the lens's focal length. In addition, the low transparency of the PTFE membrane worsens the situation. Thus, culture membranes to improve the use of inverted microscopes for live imaging of slice cultures are needed.

Here, we report a novel method for live imaging of slice cultures using a collagen membrane primarily consisting of collagen type I, which has a high cellular affinity. Collagen membranes are thinner and have a higher optical transparency than PTFE membranes, making them suitable for live imaging of slice cultures using an inverted microscope.

## 2 METHODS

### 2.1 Animals

Experiments were performed with the approval of the animal experiment ethics committee at the University of Tokyo (approval number: P29‐10) and according to the University of Tokyo's guidelines for the care and use of the laboratory animals. Experiments were conducted using postnatal day 6 (P6) C57BL/6J and CX3CR1^GFP/+^ pups. The mice were housed under controlled temperatures and light schedule (23‐25ºC and a 12‐hour light/dark cycle) and given unlimited access to food and water.

### 2.2 Slice culture

The preparation and maintenance of slice cultures, including culture media, were performed as previously described.^8^ Horizontal entorhinohippocampal slices (400‐µm‐thick) were placed on PTFE membrane filters with a published pore size of 0.45 µm (JHWP02500; Millipore) or collagen membrane filters (Kanto Chemical Co., Inc) which were then placed into donut plates (Hazai‐Ya).^2^ Collagen membranes are mainly made of bovine collagen type I. White support film was used to prevent collagen membranes from curling up. Collagen membranes can be directly purchased from Kanto Chemical Co., Inc.

### 2.3 Scanning electron microscopy

Collagen membranes were fixed in 2.5% glutaraldehyde and dehydrated using increasing concentrations of ethanol. PTFE and collagen membranes were mounted on carbon tape, sputter coated with platinum‐palladium, and examined under a scanning electron microscope (S‐4500; Hitachi) using a 10 kV accelerating voltage.

### 2.4 Membrane thickness

PTFE and collagen membranes were hydrated with Milli‐Q water. After hydration, membrane thickness was measured using a micrometer (CLM1‐15QM).

### 2.5 Optical transparency

PTFE and collagen membranes were dissected and hydrated with Milli‐Q water. Membranes were placed on glass slides (S0990490; Matsunami Glass), and cover glasses were placed over the membranes (C024401; Matsunami Glass). Transparency was measured using a spectrophotometer (U‐3310; Hitachi).

### 2.6 Immunohistochemistry

**FIGURE 2:**
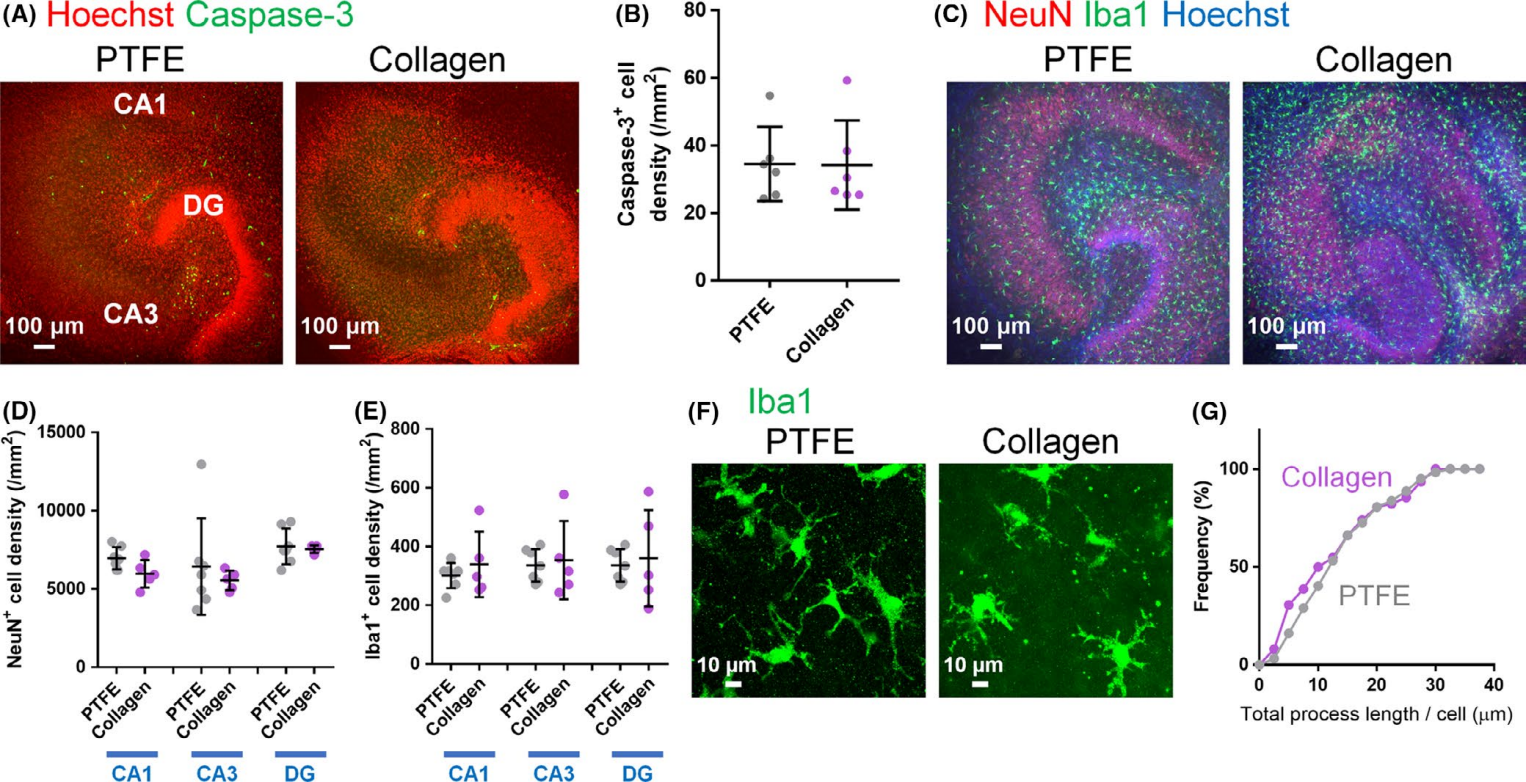
A, Representative images of the entorhinohippocampal slice cultures at 7 d in vitro (DIV) immunostained for caspase‐3. Nuclei were traced with Hoechst. B, The density of caspase‐3‐positive cells cultured on PTFE and collagen membranes. *P* > .05 vs PTFE membranes; Student's t test, n = 6 slices. Data represent mean ± SD. C, Representative images of the hippocampal slice cultures at 14 DIV immunostained for NeuN and Iba1 and DNA counterstained with Hoechst. D, E, The density of NeuN‐positive cells D, and Iba1‐positive cells E, at 14 DIV *P* > .05 vs PTFE membranes; Student's *t* test, n = 5‐7 slices. Data represent mean ± SD. F, Representative images of hippocampal slice cultures at 14 DIV immunostained for Iba1. G, Cumulative distribution of total microglial process length at 14 DIV *P* > .05 vs PTFE membranes; Kolmogorov‐Smirnov test, n = 62 processes, from 7 to 8 cells, from 3 slices

**FIGURE 3:**
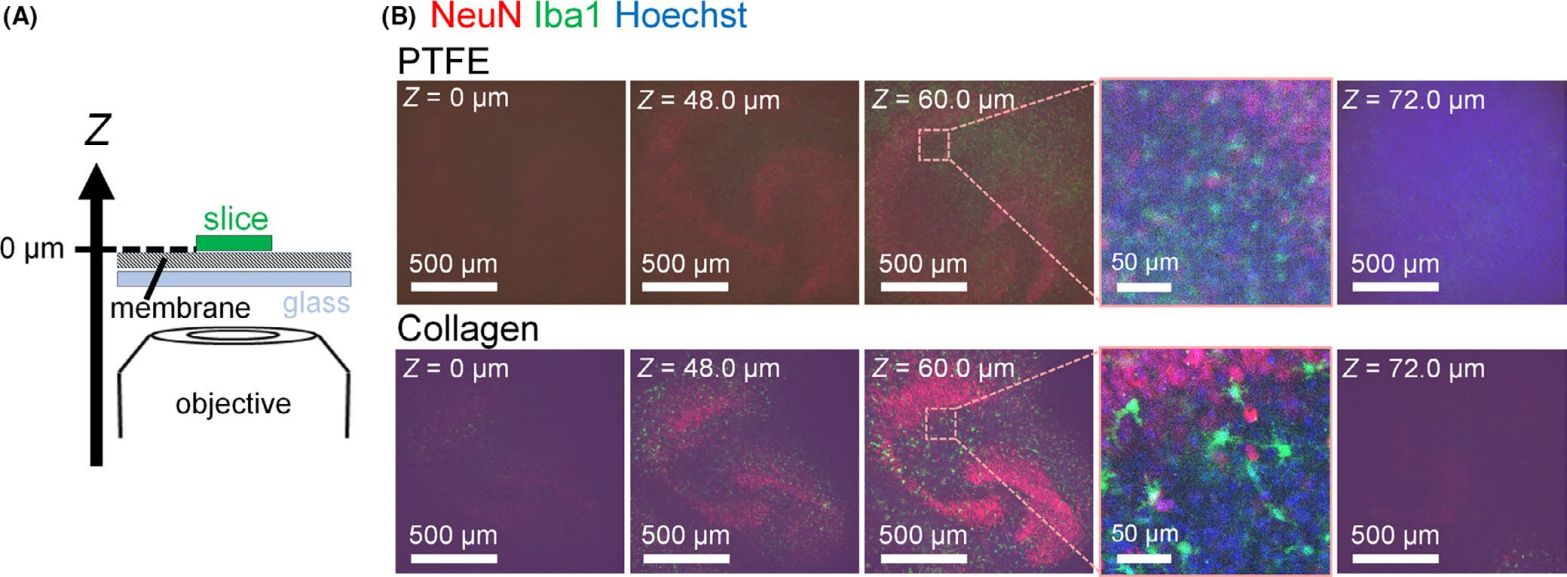
A, Schematic diagram of the live imaging setup. Immunostained slices were imaged through the culture membrane (after fixation) using an inverted microscope. The location (starting at the bottom of the slice) where staining was first observed was defined as 0 µm. B, Representative images of immunostained slice cultures at 14 DIV (NeuN and Iba1, DNA counterstained with Hoechst); 0, 48, 60, and 72 µm

Cultured slices were fixed in 4% paraformaldehyde at 4°C for 24 hours. Next, the slices were permeabilized and blocked for 1 hour using 0.3% Triton X‐100 with 10% goat serum in PBS. Primary antibody staining was performed using mouse anti‐NeuN (1:1000; MAB377; Merck Millipore), rabbit anti‐Iba1 (1:1000; 019‐19 741; FUJIFILM Wako Pure Chemical Co.), rabbit anti‐caspase‐3 (1;400; 9662; CST), and guinea pig anti‐Iba1 (1:500; 324 006; Synaptic System, Göttingen, Land Niedersachsen, Germany) followed by Alexa Fluor 488‐, 594‐, and 647‐conjugated secondary antibody staining (1:500; Thermo Fisher). Finally, the samples were embedded in PermaFluor (Thermo Fisher). Images of immunostained samples were obtained using the SpinSR10 (Olympus) confocal system with 10× (NA = 0.40) and 40× (NA = 0.95) objectives (Figure 2A‐E, Figure 3). Z‐series images were collected at 2.0 µm steps and stacked for 6 slices for caspase‐positive cell density analysis (Figure 2A,B) and 0.5 µm steps and stacked for 21 slices for microglia and neuron density analysis (Figure 2C‐E). The stacked images were analyzed using ImageJ software (NIH, Bethesda, MD, USA). Single‐plane images were shown at each depth in Figure 3.

### 2.7 Analysis of microglial morphology and dynamics

**FIGURE 4:**
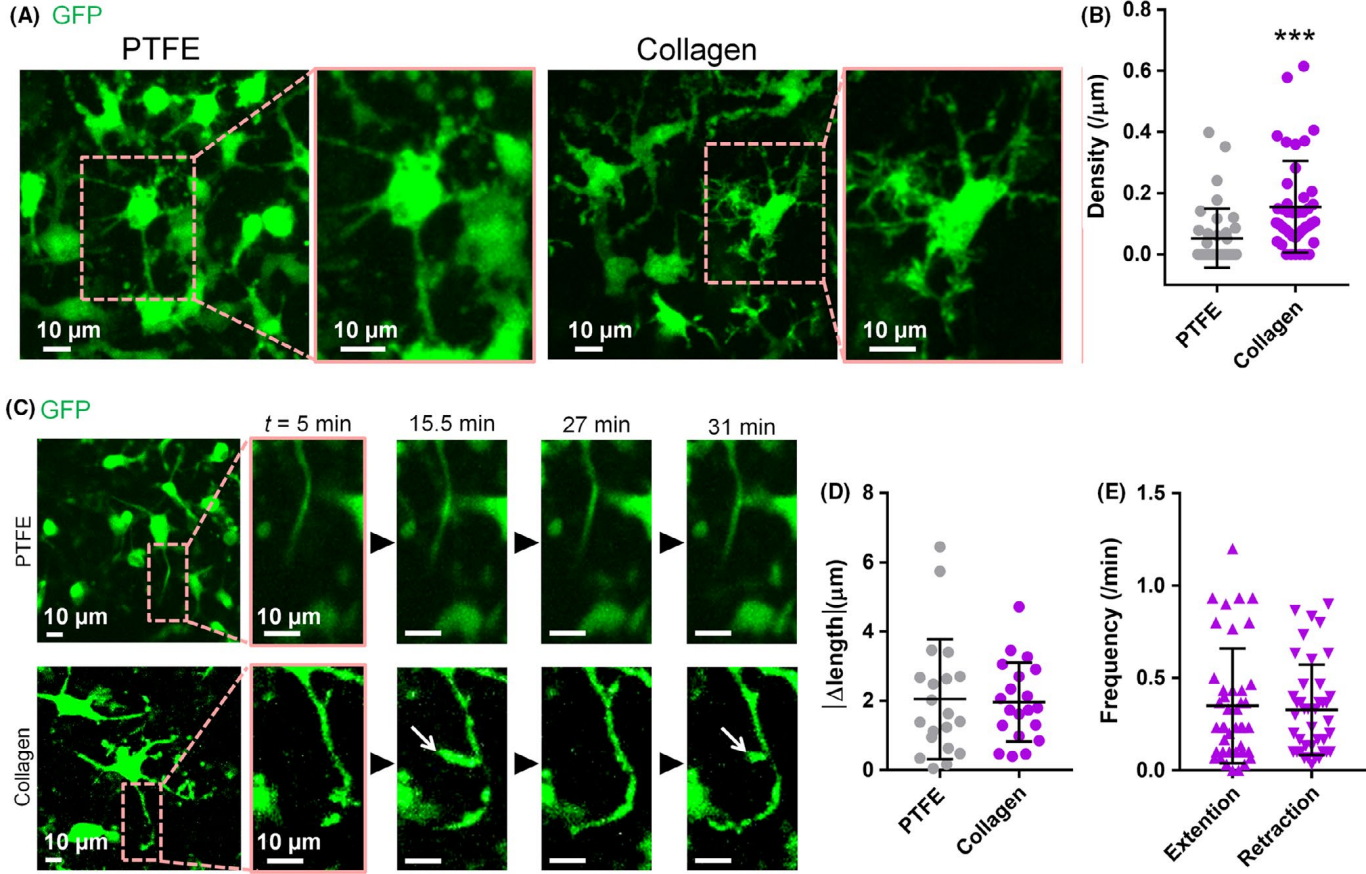
A, Representative images of slice cultures taken during live imaging at 7‐9 days in vitro (left panels). Slices were prepared from CX3CR1^GFP/+^ mice. Magnified images of the region outlined by dashed squares are shown (right panels). B, The density of short branches on each main process on PTFE and collagen membranes at 0 s. ***P < .001, Mann‐Whitney rank‐sum test, n = 38‐42 main processes from 3 slices. Data represent mean ± SD. C, Representative images of slice cultures taken during live imaging (left panels). Magnified images of the regions outlined by dashed squares are shown (right panels). Short branches of microglial processes were observed in slice cultures on collagen membranes (white arrows). D, The change in length of microglial short branches on PTFE and collagen membranes from 0 to 30 s (images were taken every 30 s). *P* > .05, Mann‐Whitney rank‐sum test, n = 20 short branches, from 8‐14 cells, from 2 slices. Data represent mean ± SD. E, Frequency of extension and retraction of short branches (slice cultures on collagen membranes) during live imaging (30 min). n = 39‐42 main processes, from 10 cells, from 3 slices. Data represent mean ± SD

For the analysis of microglial morphology and dynamics, slice culture specimens were observed using the SpinSR10 (Olympus) confocal system with a 30× (NA = 1.05) objective. Z‐series images were collected at 1.0 µm steps and stacked for 21 slices. For time‐lapse imaging, slice cultures were maintained in a humidified chamber under 37ºC and 5% CO_2_ conditions and the images were obtained at 30 second intervals. The stacked images were analyzed using ImageJ software (NIH). For the quantification of total processes (main processes and short branches) length, confocal images were obtained after immunohistochemistry (Figure 2F,G). For the quantification of density and dynamics of short branches (Figure 4), confocal images were obtained in time‐lapse imaging.

## 3 RESULTS

The membrane microstructure of both PTFE and collagen membranes was investigated using scanning electron microscopy (Figure 1B). We observed that both PTFE and collagen membranes were semiporous, allowing culture medium to infiltrate into the slice cultures. Collagen membranes were also significantly thinner than PTFE membranes (Figure 1C). These properties contributed to the significantly higher transparency of collagen versus PTFE membranes that was observed at all wavelengths (between 400 and 800 nm; Figure 1D).

To determine whether collagen membranes would be suitable for brain slice cultures, mouse entorhinohippocampal slices were cultured on both PTFE and collagen membranes. First, cell death was examined using caspase‐3 immunostaining at 7 days in vitro (DIV) (Figure 2A). There were no significant differences in the density of caspase‐3 staining between slice cultures on PTFE or collagen membranes (Figure 2B). Next, the density of NeuN‐positive neurons and Iba1‐positive microglia was examined at 14 DIV (Figure 2C). There were no significant differences in the density of neurons or microglia between slice cultures on PTFE or collagen membranes (Figure 2D,E). To evaluate the influence of membrane type on microglial morphology, we examined the length of total microglial process at 14 DIV (Figure 2F,G). Total microglial processes were defined as main process and short branch. To analyze total microglial processes, the main process of microglia was fi/rstly determined. The main process of microglia was defined as a process that directly emanated from the soma and possessed the longest length from the emanating point to the tip compared to the other processes that emanated from the same soma. Then, the branches emanated from main processes were defined as short branches. There was not a significant difference in total microglial process length between slice cultures on PTFE or collagen membranes (Figure 2G). These results suggest that the cellular conditions in slice cultures on collagen membranes were comparable to those on PTFE membranes and collagen membranes can be used to culture entorhinohippocampal slices.

Next, we examined whether collagen membranes enable the observation of deep regions in slice cultures. NeuN and Iba1 immunostained slice cultures were examined using an inverted confocal microscope (Figure 3A). Viewing the slice cultures from the bottom, the first detection of NeuN, Iba1, and Hoechst staining was defined as 0 µm (Figure 3A). Immunosignals were visible at deeper regions in slice cultures on collagen membranes than on PTFE membranes (Figure 3B). Moreover, immunosignals were more clearly visible (at any depth) in slice cultures on collagen membranes compared to those on PTFE membranes (Figure 3B). This observation was most notable at 60.0 µm. These data demonstrate that collagen membranes are suitable to detect immunosignals in deep regions of slice cultures.

Finally, live imaging of slice cultures on PTFE or collagen membranes was conducted using an inverted confocal microscope. To visualize the morphology of microglial cells, slice cultures were prepared from the brains of CX3CR1^GFP/+^ mice. Microglia in these mice have been engineered to express green fluorescent protein (GFP). The short branches that emanated from the main process of microglia in slice cultures on collagen membranes were presented (Figure 4A,C). The density of visible short branches on each main process on collagen membranes was higher than PTFE membranes at 0 seconds (Figure 4B). The movements of these short branches were followed during live imaging (Figure 4C, arrows). Though the density of visible short branches were significantly lower in slice cultures on PTFE membranes than those on collagen membranes, the dynamics of visible short branches were comparable between collagen and PTFE membrane conditions (Figure 4D). Additionally, we found that microglia in slice cultures on collagen membranes continuously extended and retracted the short branches (Figure 4E). Thus, collagen membranes allowed the observation of changes in small cellular structures in deep regions of cultured slices during live imaging using an inverted microscope.

## 4 DISCUSSION

In this study, we examined whether collagen membranes would be useful for live imaging of cellular structures in slice cultures using an inverted microscope. We found that cells in slices cultured on collagen membranes were healthy and that cellular structures could be observed in detail even in deep regions.

Organotypic slices have been usually cultured on PTFE membranes with a pore size of 0.45 μm. Because the size of pores in the membrane determines which substrates can diffuse into the slice for their survival, it can be predicted that PTFE membranes with a pore size of 0.45 μm allow the penetration of nutrients such as salts, glucose, amino acids in essential media and growth factors, adhesion molecules, hormones, lipids, and minerals in serum.

From our scanning electron microscopy images (Figure 1B), it was difficult to determine the actual pore size in collagen membrane because the collagen fibers had a mesh‐like structure and highly overlapped. A previous study reported that the average pore sizes of collagen membrane used in our experiments were about 1‐2 μm using scanning electron microscopy, which is larger than that of the PTFE membrane (0.45 μm).^9^ If the pore size of collagen membrane is actually 1‐2 μm, the nutrients required for cell survival can be diffused into slices on the collagen membrane. Though the possibility that the pore size of the collagen membrane is smaller than 1‐2 µm cannot be excluded, our findings suggested that the degree of cell death (Figure 2B) and the density of neurons and microglia (Figure 2D,E) were comparable between slice cultures on PTFE membranes and collagen membranes. Thus, it is predicted that the necessary nutrients were supplied to slice cultures on collagen membranes.

Collagen membranes enabled live imaging of short branches of microglia. It has been reported that the morphology of microglia at the top and bottom surfaces of slice cultures is different from that observed in vivo, while microglia in the center region are morphologically similar to microglia in vivo.^6^ Therefore, the center region of the slice culture should be used for microglial morphology studies. In the present study, we found that the use of collagen membranes was suitable for this purpose. While we only studied the kinetics of microglial short branches, other cellular microstructures such as neuronal spines or organelles may be detected in live imaging using this method.

Collagen is a major extracellular matrix protein that supports cellular development, differentiation, and morphology in vivo^10^, ^11^ and has long been used as a cell culture dish coating material.^12^ Neurons cultured on collagen gel‐coated glass slides are known to survive well.^11^ Many studies have suggested that cells cultured on collagen gel‐coated glass or plastic dishes survive better than cells cultured on uncoated dishes.^13^, ^14^ Such results suggest that collagen membranes may have a higher affinity for cultured slices compared to PTFE membranes. It would be interesting to develop slice culture membranes coated with extracellular matrices other than collagen, such as laminin or proteoglycan, or a mixture of these matrices. Membranes that are tailor made to best suit the specific aims of each experiment would expand the application of slice cultures.

## CONFLICT OF INTEREST

Authors declare no conflict of interest.

## AUTHOR CONTRIBUTIONS

AO conducted the experiments, analyzed the experimental data, and wrote the manuscript. TA helped prepare slice cultures. RK designed and planned the project and wrote the manuscript. YI discussed the results and commented on the manuscript.

## ANIMAL STUDIES

All experiments were performed with the approval of the animal experiment ethics committee at the University of Tokyo and according to the University of Tokyo's guidelines for the care and use of laboratory animals.

## DATA REPOSITORY

The data that support the findings of this study are available in the supplementary material of this article.
